# Lens subluxation combined with parry-romberg syndrome: case report

**DOI:** 10.1186/s12886-024-03457-y

**Published:** 2024-05-13

**Authors:** Yating Tang, Qinghe Jing, Yongxiang Jiang, Yi Lu

**Affiliations:** 1 Department of Ophthalmology and Eye Research Institute, Eye and ENT Hospital, Fudan University, 200031 Shanghai, China; 2 NHC Key Laboratory of Myopia (Fudan University), Key Laboratory of Myopia, Chinese Academy of Medical Sciences, 200031 Shanghai, China; 3 Shanghai Key Laboratory of Visual Impairment and Restoration, 200031 Shanghai, China

**Keywords:** Lens subluxation, Parry-romberg syndrome, Cataract, Progressive hemifacial atrophy, PHA

## Abstract

**Background:**

Parry-Romberg syndrome (PRS) is a rare progressive degenerative disorder of unknown etiology. Here we report a rare case of PRS combined with lens subluxation in Eye and ENT hospital of Fudan University, Shanghai. To our knowledge, it is the first reported case of PRS combined with lens subluxation that has been managed surgically with phacoemulsification and CTR placement and IOL implantation in Shanghai.

**Case presentation:**

A 60-year-old woman was referred for “right visual blur for 2 years” and had persistent right facial paralysis of unknown etiology since the age 12. She had right facial muscle atrophy and paralysis. Eye examination also showed the right eyelid pseudoptosis, enophthalmos, age-related cataract combined with lens subluxation existed in the right eye. The patient was diagnosed as age-related cataract and lens subluxation in the right eye and progressive hemifacial atrophy (Parry-Romberg syndrome). We conducted a combined phacoemulsification, IOL and CTR implantation and pupilloplasty surgery for the patient under general anesthesia and the postoperative UCVA was 20/30 and remained for 1 year’s follow up.

**Conclusions:**

Here we reported a rare case of PHA combined with lens subluxation in China. After appropriate eye surgery, the patient achieved satisfying vision result in the right eye.

## Background

Progressive hemifacial atrophy (PHA), also known as Parry-Romberg syndrome (PRS), is a rare, acquired dysfunction syndrome characterized by painless progressive facial atrophy on one side, involving the dermis, subcutaneous tissue, muscle and bone [1, 2]. PHA is also combined with other diseases such as eyes, heart, autoimmunity, infection, etc. Previous papers have reported many eye diseases combined with PHA including the enophthalmos, pseudoptosis, eyelid retraction, ciliary body and retinopathy. However, PHA combined with lens disease was rarely reported before, especially for the lens subluxation surgical treatment [3–6]. Here we reported a rare case of PHA combined with lens subluxation in Eye and ENT hospital of Fudan University, Shanghai. To our knowledge, it’s the first reported case of PRS combined with lens subluxation that has been managed surgically with phacoemulsification and CTR placement and IOL implantation in Shanghai.

## Case presentation

A 60-year-old woman, who complained of “right visual blur for 2 years”, came to our clinics of Eye and ENT hospital of Fudan University, Shanghai on 4 Apr. 2020. The patient had undergone combined cataract extraction and intraocular lens (IOLs) implantation surgery in her left eye in local hospital in 2012, and the IOL was removed in 2015 due to IOL dislocation. She had a history of uterine fibroids surgery in 2012. At the age of 12, she had persistent right facial paralysis of unknown etiology and received facial neurotrophic drug injections (specifically unknown) in local hospital. Then the right side of her face showed progressive atrophy. She did not have history of ocular trauma, infection and local or systemic allergy. Neither did she have myopia, diabetes, hypertension or other diseases. The patient had no family history of genetic diseases.

**Fig. 1:**
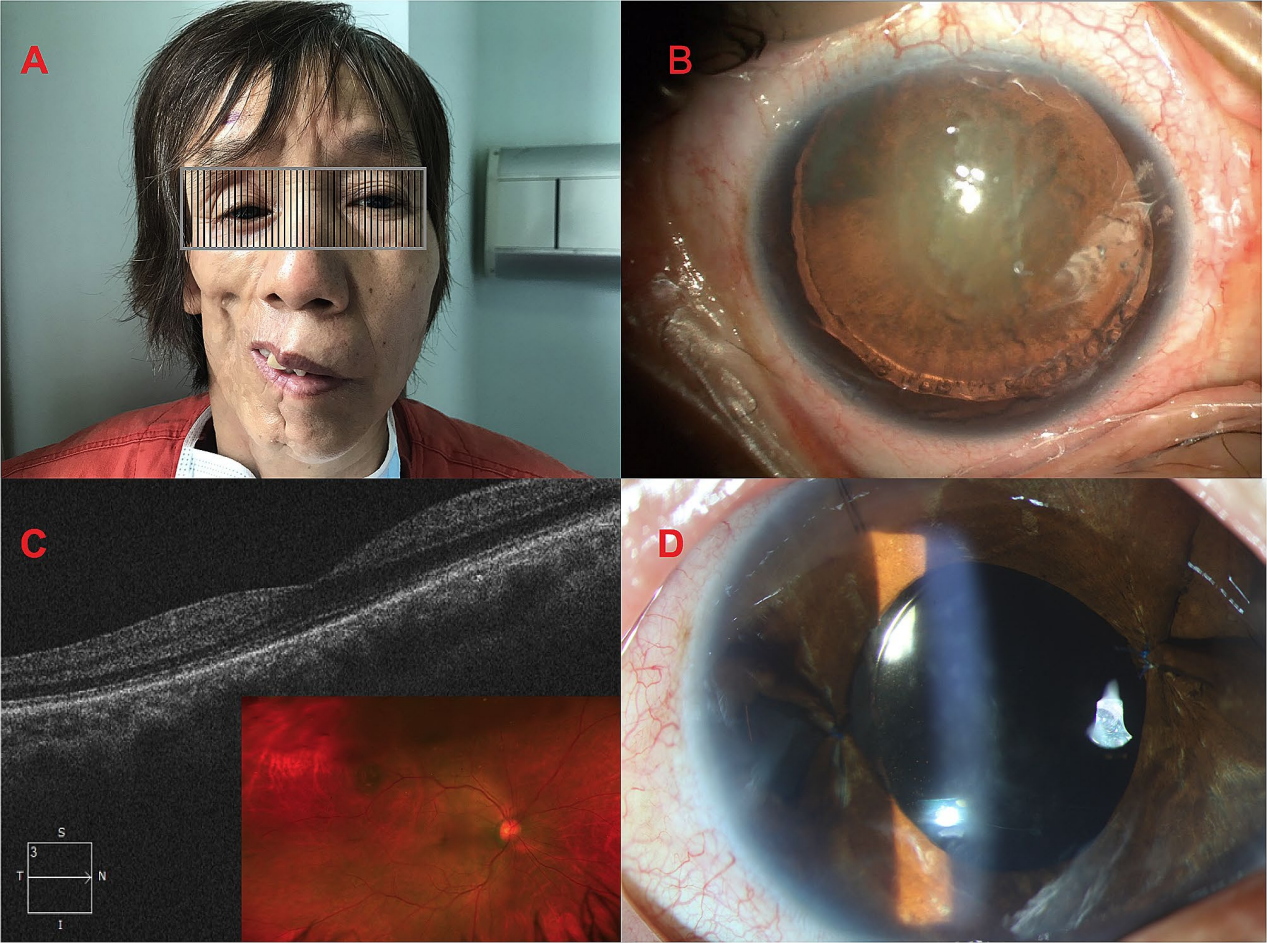
**A**. Right facial muscle atrophy with right facial paralysis. **B**. Slit lamp photo of the right eye preoperatively. **C**. Fundus photo and OCT of the right eye. **D**. Slit lamp photo of the right eye postoperatively

The patient was examined comprehensively. She had an obvious right facial muscle atrophy with right facial paralysis. Her mouth and nose were deviated toward the right side of hemifacial atrophy (Fig. 1A). Her best corrected visual acuity was counting fingers in both eyes. The intraocular pressure was normal in her both eyes. She had pseudoptosis eyelid and enophthalmos in the right eye. No vertical and horizontal motility restriction were found in both eye. The pupils were dilated about 10 mm diameters with iris atrophy in both eyes. The right lens was opaque with Lens Opacity Classification System III grading cortical grade 3 nuclear grade 3 and postcapsular grade 4. The zonular fiber was loosened and elongated by nearly 220° (lens subluxation) in the right eye (Fig. 1B). The fundus examination was normal in the right eye. Her left eye was aphakic with obvious optic nerve atrophy (C/D 1.0) and disorganized macula area. Fundus OCT indicated that the macular structure was normal in the right eye (Fig. 1C). Her head MRI report showed several ischemic foci in the brain, empty sella and cerebral arteriosclerosis changes. Based on the above examination, the diagnosis was age-related cataract combined with lens subluxation in the right eye, aphakic eye and optic nerve atrophy in the left eye, and progressive hemifacial atrophy (Parry-Romberg syndrome).

After communication and the patient’s admission, the other cataract surgery relevant examinations were performed. We conducted a combined phacoemulsification, IOL and CTR implantation and pupilloplasty surgery for the patient under general anesthesia. The operation was very successful. The patient was satisfied with the UCVA 20/30 on the first day post-operation. The cornea was clear, anterior chamber was quiet, pupil diameter was about 5 mm (Fig. 1D). We followed her for 3 months, 6 months and 12 months, respectively. No complication was found and the UCVA and BCVA remained 20/30.

## Discussion and conclusions

PHA was first reported by Parry in 1825 and described in details by Romberg in 1846. However, it was not until 1871 that Eulenberg gave the disease its current nomenclature: progressive hemifacial atrophy. PHA is a sporadic and rare dysfunction syndrome, with an incidence rate of 1: 700,000, characterized by painless progressive facial atrophy and involved skin, subcutaneous tissue, muscle and bone. PHA slowly progresses between the ages of 2 and 20 and occurs frequently in females with a male to female ratio 1:4. There is no difference in the probability of left or right facial involved. The disease can continue to progress for 7–9 years (the active phase) and then enters the stable phase, which often affects the development of the face and head [7].

The pathogenesis of Parry-Romberg syndrome is not yet fully understood. It may be caused by the hyperactive cervical sympathetic nerve caused by immune regulation and central nervous system disorders. In addition, there are many hypotheses, such as trauma, infection, autoimmune system disorder hypothesis, hyperactivity of the brain stem sympathetic centers, cervical sympathetic dysfunction, scleroderma hypothesis, but neither of them can fully explain the pathogenesis of the disease. Trauma or surgery is sometimes a cause of PHA in 24–34% of patients. Some clinical have reported familial PHA, but there is yet no sufficient evidence to prove the disease is a special genetic disease [8].

PHA is a chronic progressive disease that mostly affects one side, and in some cases, bilateral cases have also been reported [9]. According to its typical hemilateral atrophy, PHA can be diagnosed clinically. The initially manifestation of PHA was atrophy of the skin and subcutaneous tissues, which can involve the skin and muscles of the mouth, nose, ears, and cheeks. The disease is often limited to the trigeminal nerve area and generally does not cross the midline, which eventually leads to craniofacial asymmetry, often accompanied by neurological effects, such as epilepsy, migraine, facial paresthesia, trigeminal neuralgia, and hyperpigmentation. CT and MRI can detect intracranial lesions. The most common changes are brain parenchymal calcification, white matter abnormalities, and brain atrophy [10]. Most patients with epilepsy have abnormalities in the electroencephalogram and the corresponding frontal and temporal lobes or extensive imaging abnormalities in the epilepsy.

In addition, PHA may also be associated with eye and heart abnormalities, scleroderma, involvement of autoimmune system, infection, endocrine, oral and maxillofacial systems. Stone et al. [11] investigated 205 PHA patients and found that 11% had epilepsy, 52% had facial neuralgia, 46% had eye or vision disorders, and 46% had anxiety or depression. Many cases are also associated with other autoimmune system diseases, such as vitiligo (17%), thyroid disease (10%), systemic sclerosis (5%), inflammatory bowel disease (5%), joint stiffness (2%) and systemic lupus erythematosus (2%)^11^.

Ocular diseases occur in 10–35% of PHA patients, mainly on the side ipsilateral to facial atrophy, but the opposite eye may also be affected [8]. Currently, most ocular findings are described in case reports. The periorbital cavity is often affected. The most frequent sign is enophthalmos due to atrophy of the retrobulbar fatty tissue. In addition, pseudoptosis and eyelid retraction may also occur. Ocular manifestations can develop before, during or after facial atrophy. Eye disorders include cornea, sclera, ciliary body and retinal changes such as corneal epithelial and stroma or endothelial cell count disorder, corneal nerve reduction, endothelium reduction, stroma deposition, sclera dissolution, iris atrophy. The reported combined uvea disorders and retinopathy [8] include anterior, middle and pan uveitis, Fuchs heterochromatic iridocyclitis, retinal vasculitis, retinal pigment epithelial changes, retinal edema, retinal detachment, Coats disease, focal Choroidal retinal atrophy, central retinal artery occlusion. Neuro-ophthalmological diseases include oculomotor nerve palsy, optic nerve atrophy, optic neuritis, horner syndrome, dilated pupil, addie’s pupil and strabismus. Optic nerve papillitis and optic neuroretinitis may occur. In some cases of PHA, there may be anisocoria on the same side of the lesion, such as dilated or miotic pupils. In addition, oculomotor nerve palsy and extraocular muscle fibrosis may cause diplopia and strabismus [12].

This case is the first reported patient of PHA associated with lens subluxation in China. The patient had typical signs of facial atrophy of the right side and the disease is persistent, the PHA diagnosis is clear. Moreover, she had poor vision in bilateral eyes, right lens opacity associated with lens subluxation of the right lens, atrophy of bilateral iris and large pupil about 10 mm in both eyes. Also, she had a surgery history of cataract extraction and IOLs implantation surgery in her left eye, but then the IOL was removed three years later due to IOL subluxation in local hospital. Based on the eye examination and her surgical history, we speculated the ophthalmological manifestations (lens subluxation, iris atrophy and dilated pupils) might existed in both eyes.

CT and MRI are two most common modalities used in the diagnosis of the neurological features in PHA. Most of the imaging findings were ipsilateral. A common MRI findings is ipsilateral white-matter high signal intensity, with occasional hyperintensities in the gray matter as well [9]. Other MRI findings such as intracerebral atrophy [13], mild cortical thickening [13, 14], dense mineral deposition or calcification [13] were also found in some cases. However, these neurological findings were not found in our case. Her head MRI showed several ischemic foci in the brain, empty sella and cerebral arteriosclerosis changes, which was not specific features of PHA.

Little is known about the efficacy of agents using to treat this disease because no randomized controlled trial was conducted. Suggested treatment include immune inhibitors, local or systemic steroids, vitamins [8]. Sympathectomy appears to have halted disease progression in some cases. Symptomatic treatment strategies are often used for comorbid diseases, such as facial plastic surgery, neurological and eye treatment.

Ophthalmological treatment focus on the stabilization of ocular complications or rehabilitation of vision function. External eye and facial cosmetic surgery may be successful to reconstruct natural face and treat enophthalmos and pseudoptosis. External eye muscle surgery and frontalis suspension may be indicated to correct strabismus and ptosis [15]. Uveitis is treated with topical or systemic steroid and mydriasis, in some cases, immunosuppressive therapy may also need. Retinal arteriolar leakage may be treated laser photocoagulation and in some cases pars plana vitrectomy may be necessary because of retinal exudates and retinal detachment. Lens surgery is necessary in cases of cataract or lens subluxation. In this case, the patient’s lens subluxation was close to 220° with clinical cataract, so we adopted the surgical method of phacoemulsification, a CTR implantation combined with IOL implantation, and a good vision outcome was achieved post operation and for 1 year’s follow-up.

In conclusion, PHA is a rare progressive degenerative disorder of unknown etiology. Besides characteristic facial hemiatrophy, PHA often presents with a variety of ophthalmological manifestations. Here we reported a rare case of PHA combined with lens subluxation in Shanghai. After appropriate eye treatment, the patient achieved satisfying vision result in the right eye.

## Data Availability

**statements**: All data generated or analyzed during this study are included in this published article.
